# Notch1 and Major Vault Proteins Modulate Temozolomide Resistance in Glioblastoma

**DOI:** 10.1111/jcmm.70474

**Published:** 2025-03-18

**Authors:** Cengiz Tuncer, Ceyhan Hacioglu

**Affiliations:** ^1^ Faculty of Medicine, Department of Neurosurgery Düzce University Düzce Turkey; ^2^ Faculty of Medicine, Department of Medical Biochemistry Düzce University Düzce Turkey; ^3^ Faculty of Pharmacy, Department of Biochemistry Düzce University Düzce Turkey

**Keywords:** glioblastoma multiforme, major vault protein, notch, temozolomide

## Abstract

The development of resistance to chemotherapy in the case of aggressive glioblastoma multiforme (GBM) presents a significant treatment challenge. Dysregulation of the Notch signalling pathway promotes tumour proliferation in GBM cells. This study was that targeting the Notch signalling pathway could be a potential therapeutic approach for GBM. Initially, temozolomide‐(TMZ)‐resistant GBM cells were generated, and the effect of Notch1 on the expression of multiple resistance proteins within these cells was investigated. Subsequently, the expression of Notch‐1 in GBM cells was reduced using siRNA. Results revealed a significant reduction in TMZ sensitivity in TMZ‐resistant GBM cells, accompanied by a substantial increase in the expression of major vault protein‐(MVP), O6‐methylguanine‐DNA‐methyltransferase‐(MGMT), and ATP‐binding‐cassette transporter‐G2‐(ABCG2). Furthermore, TMZ‐resistant U87‐R and U251‐R cells exhibited higher proliferation rates compared to their parental control cells (U87 and U251). Additionally, we observed that downregulating Notch‐1 signalling inhibited the proliferation of TMZ‐resistant U87‐R and U251‐R cells. This downregulation led to the inactivation of MGMT, ABCG2, and MVP. Importantly, it increased chemosensitivity to TMZ, particularly by downregulating MVP expression. Consequently, Notch1 could serve as a potential therapeutic target for GBM cells and may be effective in preventing TMZ resistance by targeting MVP, as well as MGMT and ABCG2 in GBM cells.

## 1 Introduction

Glioblastoma multiforme (GBM) stands as one of the most formidable cancer types, characterised by a median survival period of approximately 1 year [1]. GBM presents unique challenges in terms of treatment due to its specific location, highly aggressive biological characteristics, and extensive, invasive growth [2]. Despite advancements in surgical and radiation techniques, along with the utilisation of various chemotherapeutic agents, the quest for a cure for malignant gliomas remains a formidable undertaking [3]. The limited effectiveness of current therapeutic methods underscores the resilience of GBM cells to cytotoxic treatments, even in laboratory settings. Furthermore, the short interval before tumour recurrence in GBM patients suggests that tumourigenic cells can withstand treatments without suffering significant harm [4].

The Notch receptor plays a pivotal role in a highly conserved signalling pathway that holds significant importance in both the development of the central nervous system and the process of malignant transformation [5]. The Notch receptors belong to a category of transmembrane proteins, including Notch1, Notch2, Notch3, and Notch4. The initiation of Notch‐1 signalling occurs through the formation of a complex resulting from the interaction between ligands and receptors located on the surface of neighbouring cells [6]. Subsequently, Notch1 is cleaved by an enzyme known as γ‐secretase, and the resulting Notch‐1 intracellular domain (NICD) is translocated from the cell membrane into the cell nucleus. Inside the nucleus, NICD regulates specific target genes, including those in the hairy enhance of split (Hes) family. There is a growing body of evidence suggesting a close connection between Notch1 signalling and chemotherapy resistance in various cancer types [7, 8]. Prior research has indicated that Notch1 possesses the capacity to regulate ABC transporters, proteins involved in cell death processes, thereby influencing the sensitivity of tumour cells to chemotherapy drugs [9]. For example, Zhang and colleagues demonstrated that targeting Notch1 could modulate cell sensitivity to docetaxel, a chemotherapy drug [10]. Recent research studies have explored the expression patterns of Notch1 in gliomas, yielding diverse conclusions regarding its impact on tumour progression and prognosis [11, 12]. Notably, it has been documented that Notch1 displays abnormal expression in gliomas of all grades, and overexpression of Notch1 is linked to a poorer survival outcome [13]. However, the molecular mechanisms underlying the oncogenic and anti‐oncogenic activities of Notch1 have not been fully understood.

The Major vault protein (MVP) serves to regulate chemosensitivity in cancer metabolism, a recurrent challenge encountered during cancer treatment [14]. MVP assumes a central role within the vault complex, a ribonucleoprotein structure renowned for its characteristic hollow, barrel‐like morphology. This complex is responsible for the efflux of drugs from the cell nucleus, sequestering them within cytosolic vesicles [15]. Extensive research has revealed a pronounced increase in MVP levels within cancer cells that develop resistance to chemotherapeutic agents following treatment [16, 17]. In the context of brain tumours, one contributing factor to the limited efficacy of chemotherapy is the presence of ABC‐binding cassette carrier proteins, actively removing the administered chemotherapeutic agents from the brain [18]. Additionally, MVP has been associated with various ABC proteins in tumour cells [19]. However, ongoing research is required to fully elucidate the specific role of this protein in GBM, particularly its contribution to drug resistance mechanisms and clinical outcomes.

In this study, our primary objective was to define the impact of the Notch1/MVP signalling pathway on the development of chemoresistance in GBM cells. Specifically, we examined how the regulation of Notch1 affects sensitivity to temozolomide (TMZ) in TMZ‐resistant GBM cells. Furthermore, we investigated whether the regulation of Notch1 and MVP expression could serve as therapeutic targets against TMZ resistance in primary cells isolated from GBM patients who had not received TMZ treatment.

## 2 Materials and Methods

### 2.1 Cell Culture Conditions

The human GBM cell lines (U87 and U251) used in this study were obtained from the American Type Culture Collection (ATCC). To ensure the provision of an optimal growth environment, these specific cell lines were cultivated in Dulbecco's Modified Eagle's Medium (DMEM). This medium was supplemented with essential constituents, including 2 mM glutamine, 10% fetal bovine serum (FBS), 100 U/mL–100 μg/mL penicillin–streptomycin. The cells were meticulously maintained in a controlled incubator, set at a temperature of 37°C, with a controlled atmosphere of 5% CO_2_.

### 2.2 Development of TMZ‐Resistant Cell Lines

To generate TMZ‐resistant GBM cell lines, we followed the procedure outlined in our previous study [20]. In a brief summary, U87 and U251 cells were subjected to repeated exposure to 200 μM TMZ every 72 h over a 3‐week period. During each 3‐day cycle, fresh culture medium with 200 μM TMZ was consistently replenished. While the majority of cells succumbed to TMZ exposure and failed to survive, a resistant minority demonstrated the capacity to withstand and sustain the proliferation of cells. These tenacious surviving cells were subsequently transferred to new culture dishes and provided an environment enriched with a 200 μM concentration of TMZ. These cell clusters, manifesting their acquired resistance to TMZ, were duly designated as the U87‐R and U251‐R cell lines.

Following the successful establishment of these TMZ‐resistant cell lines, the U87‐R and U251‐R cells were individually seeded in 6‐well plates and subjected to an overnight incubation at 37°C to ensure proper cell adhesion. Subsequently, these cells were subjected to TMZ concentrations in the range from 250 to 2000 μM for 24, 48, and 72 h.

### 2.3 RNA Interference of Notch1

We used siRNA molecules, specifically designed to target Notch1, with the sequence for siNotch1 (Santa Cruz Biotechnology) (sense 5′‐UGU CAU CCA UCA GAA CUG GGG‐3′, and antisense 5′‐CCU UGU GAC UUU UCG UAA UUA‐3′). In addition, we obtained a negative control siRNA (siCNT). Cells were transfected with either the Notch1‐specific siRNA or the non‐targeting siRNAs. One day before transfection, cells were seeded onto fresh plates, and Lipofectamine 3000 (Thermo Fisher Scientific, USA) was used for transfection in accordance with the manufacturer's guidelines. Six hours after transfection, the serum‐free medium was replaced with a new culture medium. In the following experiments, the cells were subjected to a transfection process for 48 h.

### 2.4 Cell Proliferation Analyzes

Proliferation in TMZ‐resistant (U87‐R and U251‐R) and parental (U87 and U251) cells was assessed utilising the Cell Counting Kit 8 (CCK‐8; Cat No: E‐CK‐A362), according to the manufacturer's recommended protocols. In the CCK‐8 assay, cells were allowed a 24‐h incubation period for adhesion prior to exposure to TMZ treatment for durations of 24, 48, and 72 h. Following the treatment intervals, the cells were treated with 100 μL of the CCK reagent in each well and incubated at 37°C for 60 min. Subsequently, the colour intensity was measured at 450 nm with the help of a microplate reader.

### 2.5 Enzyme‐Linked Immunosorbent Assay (ELISA) Analyzes

Notch‐1, MVP, O6‐methylguanine‐DNA methyltransferase (MGMT), ATP‐binding cassette transporter G2 (ABCG2), vimentin, and N‐cadherin were examined using ELISA kits (EHNOTCH1, MBS2703677, LS‐F12032, CSB‐E11251h, E‐EL‐H1094 and E‐EL‐H0195, respectively), following the manufacturer's instructions. The optical density (OD) values were measured at a wavelength of 450 nm using a microplate reader (Epoch, BioTek), and the absolute protein concentrations were determined based on the standard curves.

### 2.6 Quantitative Real‐Time PCR Method

Cells were lysed with radioimmunoprecipitation (RIPA) buffer (Santa Cruz Biotechnology, USA). Total RNA was extracted from cells using TRIzol reagent (Invitrogen, 12594025), following the manufacturer's guidelines. The obtained RNA was subsequently subjected to reverse transcription using the SuperScript IV One‐Step RT‐PCR System, with 1 μg of cellular RNA utilised in this process. For the amplification of complementary DNAs (cDNAs), the StepOnePlus Real‐Time PCR System was employed in combination with SYBR Green Master Mix. The relative mRNA expression levels were assessed using the $2^{-\Delta \Delta C_{t}}$ method, which involves normalising gene expression values to the internal control β‐actin. The PCR cycling conditions were as follows: a preheat step at 95°C for 15 min, a denaturation step with 45 cycles at 95°C for 20 s, and annealing/extension at 55°C for 60 s. The primer sequences were as follows: MVP forward 5′‐CAG GAT GTG TAT GTG CTG CGG‐3′, MVP reverse 5′‐GCT GGA GGC TCT TAG CTG GTC‐3′; Notch1 forward 5′‐GCT ACA ACT GCG TGT GTG TCG‐3′, Notch reverse 5′‐GTT GGT GTC GCA GTT GGA GCC‐3′; ABCG2 forward 5′‐GCG TGC GCA GAA TAT TGC CGC AGC‐3′, ABCG2 reverse 5′‐GCT GGA TGT CAA GTA GAC GCG‐3′; MGMT forward 5′‐GCC GAC AGC CAA GTT CCA CCG‐3′, MGMT reverse 5′‐CGC ACC CTG CAT CTG CAT CGA‐3′. β‐actin forward: 5′‐GTG GAC ATC CGC AAA GAC‐3′, and reverse: 5′‐AAA GGG TGT AAC GCA ACT A‐3′.

### 2.7 Western Blot Analysis

To quantify protein expression, cells were incubated with RIPA lysis buffer (89900; Thermo Scientific). Then, the extracted proteins were loaded in equal volumes into each well of the 10% SDS‐PAGE gel, and following their separation in the electrical field, the proteins were transferred to a polyvinylidene fluoride (PVDF) membrane. Subsequently, the membranes underwent a blocking step, involving immersion in a solution consisting of 0.05% Tween and 5% bovine serum albumin (BSA) in Tris‐buffered saline, for 2 h at room temperature. They were incubated overnight at 4°C with primary antibodies targeting proteins such as Notch1 (dilution 1:500; MA5‐32080), ABCG2 (dilution 100 μg/mL; sc‐58222), MVP (dilution 1:500; PA5‐82011), MGMT (dilution 1:100; PA5‐85946), vimentin (dilution 1:10000; PA5‐27231), N‐cadherin (dilution 1:1000; PA5‐29570) and β‐actin. Following the blocking step, the prepared membranes underwent an additional procedure in which they were exposed to secondary antibodies conjugated with horseradish peroxidase. Subsequently, the immunological complexes were visualised using an electrochemiluminescence kit (34,579, Thermo Scientific). For the quantitative assessment of the protein bands, the band intensities were analysed using Image J software.

### 2.8 Statistical Analysis

All experiments were conducted using GraphPad Prism 8 (GraphPad Software Inc). The data were presented as the mean ± standard deviation (SD) derived from triplicate replicates of three independent experiments. To assess significant differences in means between different groups, we utilised Student's unpaired *t*‐test for independent analyses. In cases involving multiple groups, a one‐way analysis of variance (ANOVA) was employed, followed by Tukey's post hoc tests for conducting comparisons across multiple groups. A *p*‐value of less than 0.05 was considered statistically significant.

## 3 Results

### 3.1 Expression of Multidrug Resistance Proteins and Cell Proliferation in TMZ‐Resistant Cells

**FIGURE 1:**
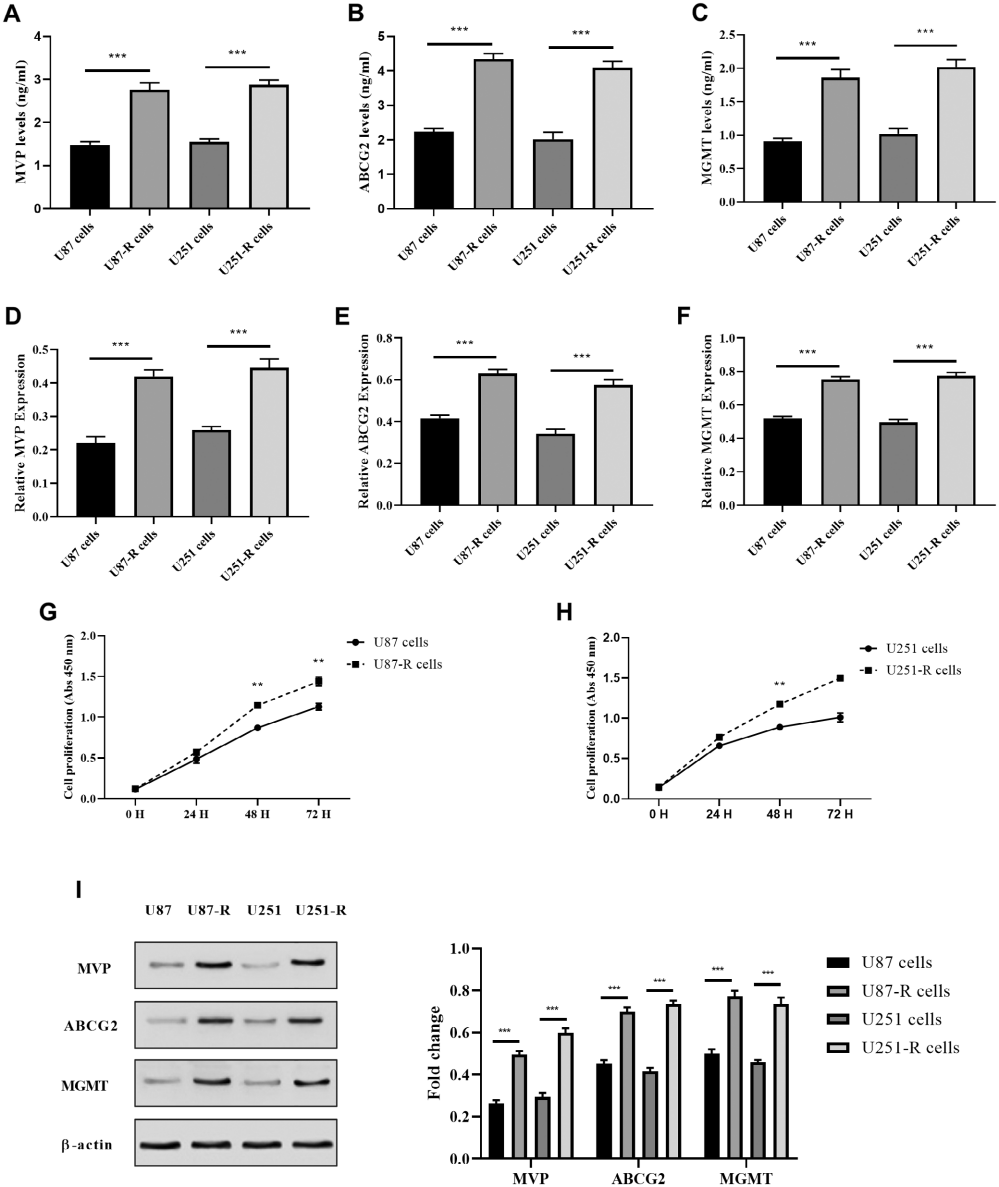
MVP, MGMT, and ABCG2 expressions increased in TMZ resistance‐GBM cells. (A) ELISA analysis of MVP levels in TMZ‐resistant and non‐resistant GBM cells, (B) ELISA analysis of ABCG2 levels in TMZ‐resistant and non‐resistant GBM cells, (C) ELISA analysis of MGMT levels in TMZ‐resistant and non‐resistant GBM cells, (D) RT‐PCR analysis of MVP mRNA levels in TMZ‐resistant and non‐resistant GBM cells, (E) RT‐PCR analysis of ABCG2 mRNA levels in TMZ‐resistant and non‐resistant GBM cells, (F) RT‐PCR analysis of MGMT mRNA levels in TMZ‐resistant and non‐resistant GBM cells, (G) CCK‐8 analysis in U87 and U87‐R cells, (H) CCK8 analysis in U251 and U251‐R cells, (I) Western blot analysis of MVP, MGMT, and ABCG2 protein levels in TMZ‐resistant and non‐resistant GBM cell. **p < 0.001, and ***p < 0.0001 compared with control cells.

TMZ is an important chemotherapy agent currently used in the treatment of GBM. However, the emergence of drug resistance poses a significant challenge to its clinical effectiveness. In this study, we conducted an experiment involving U87 and U251 cells to investigate drug resistance to TMZ. Initially, these cells were exposed to a concentration of 200 μM TMZ in the culture medium for 3 weeks. Extended exposure resulted in the development of cells resistant to TMZ, denoted as U87‐R and U251‐R cells. In line with the observed heightened resistance to TMZ, there was a significant increase in the levels of multidrug resistance‐associated proteins, which included MVP, ABCG2, and MGMT, in U87‐R and U251‐R cells when compared to parental cells (U87 and U251). This observation suggests a potential correlation between the heightened expression of these proteins and the development of resistance to TMZ. ELISA results revealed increased levels of MVP, ABCG2, and MGMT in U87‐R and U251‐R cells (*p* < 0.0001 vs. U87 and U251 cells; Figure 1A–C). Likewise, the MVP, ABCG2, and MGMT mRNA levels were notably higher in U87‐R and U251‐R cells when compared to their counterparts, U87 and U251, respectively (*p* < 0.0001; Figure 1D–F). Additionally, as shown in Figure 1I, MVP, ABCG2, and MGMT protein levels were elevated in TMZ‐resistant cells (U87‐R and U251‐R).

**FIGURE 2:**
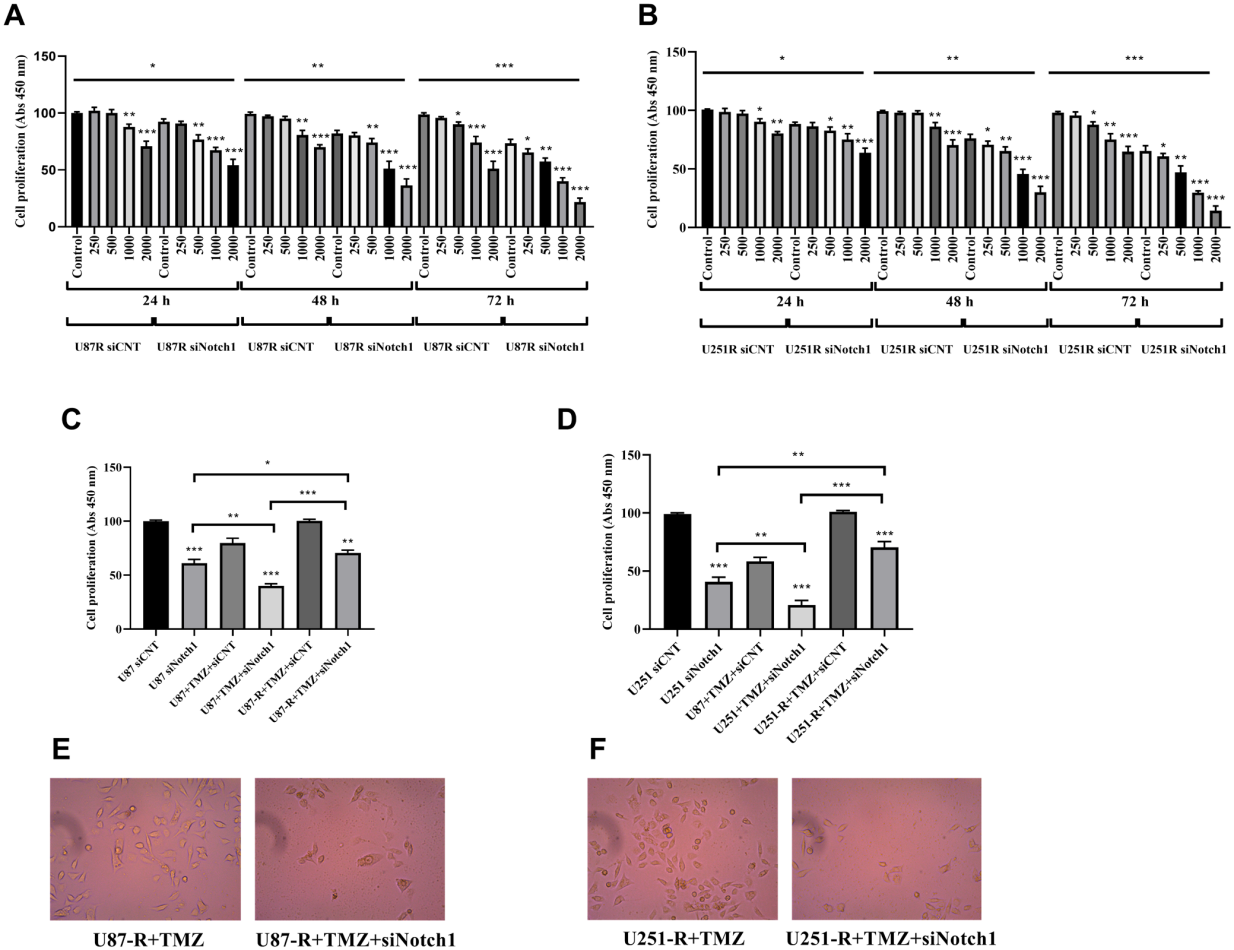
Knockdown of Notch1 reduced TMZ resistance in GBM cells. (A) CCK8 analysis of TMZ treatment (0–2000 μM) up to 72 h in U87‐R cells, (B) CCK8 analysis of TMZ treatment (0–2000 μM) up to 72 h in U251‐R cells, (C) CCK‐8 analysis in U87 and U87‐R cells, (D) CCK‐8 analysis in U87 and U87‐R cells, (E) U87‐R cells treated with 600 μM TMZ and siNotch1 for 48 h, (F) U251‐R cells treated with 600 μM TMZ and siNotch1 for 48 h. Images in E, F were viewed with a 20× objective. *p < 0.05, **p < 0.001, and ***p < 0.0001 compared with control cells.

Based on the findings from the CCK‐8 analysis, it was evident that U87‐R and U251‐R cells exhibited higher rates of proliferation at 24, 48 and 72 h when compared to U87 and U251 cells (Figure 1). Although a moderate increase was detected in the proliferation rate of U87‐R and U251‐R cells at the 24 h, no statistically significant difference was noted (*p* > 0.05 vs. U87 and U251; Figure 2G,H). However, U87‐R and U251‐R cells showed a significant increase in proliferation at 48 and 72 h compared to U87 and U251 cells (*p* < 0.001).

### 3.2 Downregulation of Notch1 Was Associated With TMZ Sensitivity

To investigate the potential role of Notch1 in promoting drug resistance in GBM cells and its interaction with other proteins associated with multidrug resistance, we conducted si‐Notch1 transfections to reduce its expression in the cells. Subsequently, we evaluated the responsiveness of GBM cells to TMZ. We also assessed how U87‐R and U251‐R cells with reduced Notch1 levels responded to TMZ by observing their reactions to TMZ treatment at three different time points (24, 48, and 72 h). Our results, as depicted in Figure 2A, revealed that the downregulation of Notch1 led to a reduction in cell proliferation, which depended on both the concentration and duration of TMZ treatment, particularly notable when treated with TMZ for 72 h in U87‐R cells. Similarly, the transfection of U251‐R cells with si‐Notch1 resulted in decreased cell proliferation, with a more pronounced effect observed at higher TMZ concentrations, as illustrated in Figure 2B. Ultimately, our findings indicate that cells with reduced Notch1 expression displayed a heightened sensitivity to TMZ in contrast to the control cells, specifically U87‐R and U251‐R cells.

To evaluate the effect of Notch1 on the proliferation of GBM cells not treated with TMZ, we treated U87, U251, U87‐R, and U251‐R cells with a previously determined potentially cytotoxic concentration of TMZ (600 μM) [20] for 48 h. As depicted in Figure 2C,D, the knockdown of Notch1 resulted in reduced proliferation in parental cells. Furthermore, it was evident that silencing Notch1 significantly inhibited cell proliferation, effectively overcoming the TMZ resistance observed in TMZ‐resistant cells. Moreover, cells in the U87‐R + TMZ, U251‐R + TMZ, U87‐R + TMZ + siNotch1, and U251‐R + TMZ + siNotch1 groups treated for 48 h are shown in Figure 2E,F. U87‐R + TMZ + siNotch1 and U251‐R + TMZ + siNotch1 cells both decreased in number and differed morphologically (cell shrinkage, cellular rolling and vacuolisation, etc.) when compared to the cells in the U87‐R + TMZ and U251‐R + TMZ groups, respectively.

**FIGURE 3:**
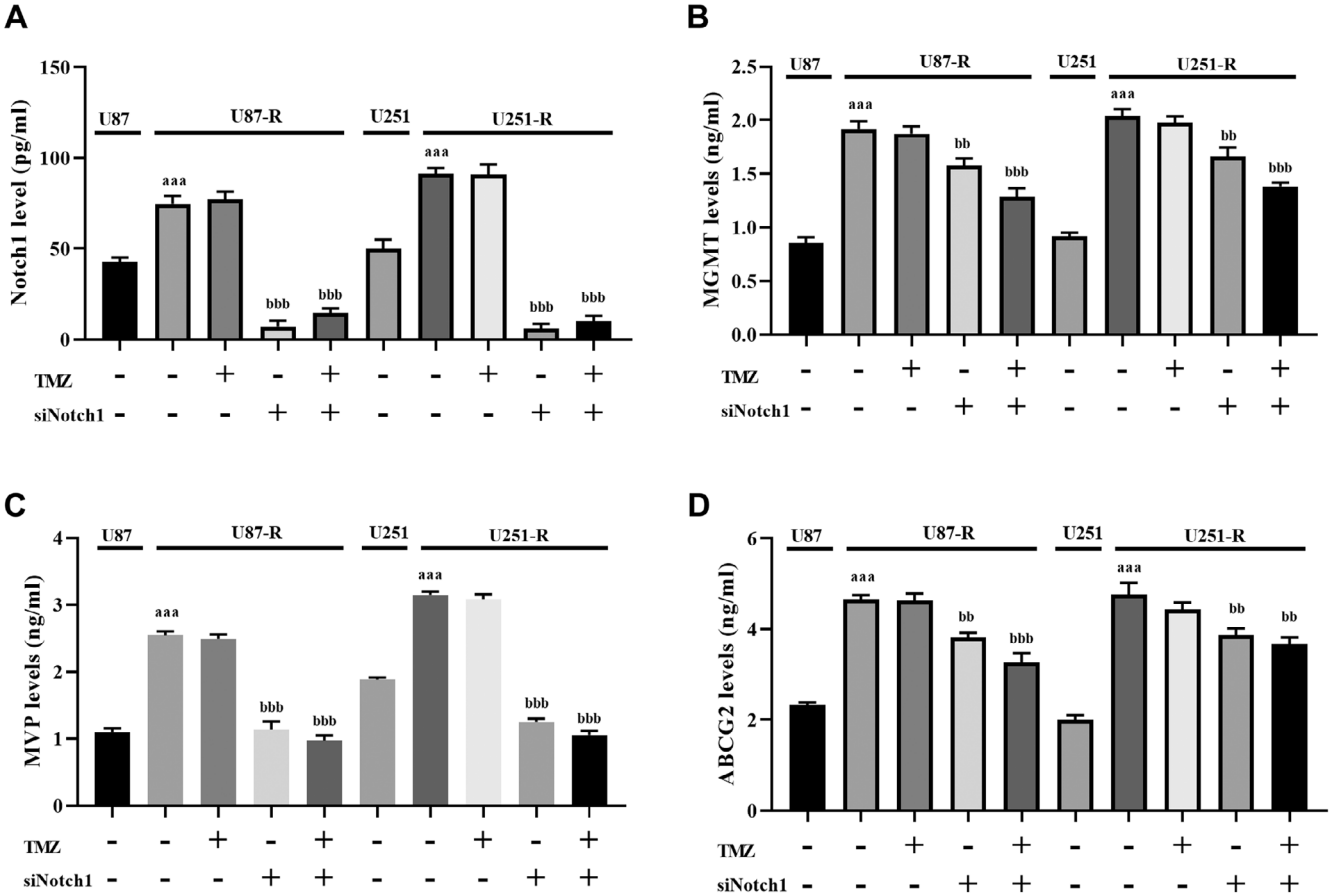
Knockdown of Notch1 reduced the multiple resistance proteins levels in GBM cells. (A) ELISA analysis of Notch1 levels in TMZ‐resistant and non‐resistant GBM cells, (B) ELISA analysis of MGMT levels in TMZ‐resistant and non‐resistant GBM cells, (C) ELISA analysis of MVP levels in TMZ‐resistant and non‐resistant GBM cells, (D) ELISA analysis of ABCG2 levels in TMZ‐resistant and non‐resistant GBM cells. ^aaa^ *p* < 0.0001 compared with TMZ‐non‐resistant GBM cells, ^bb^ *p* < 0.001 compared with TMZ‐resistant GBM cells, and ^bbb^ *p* < 0.0001 compared with TMZ‐resistant GBM cells.

**FIGURE 4:**
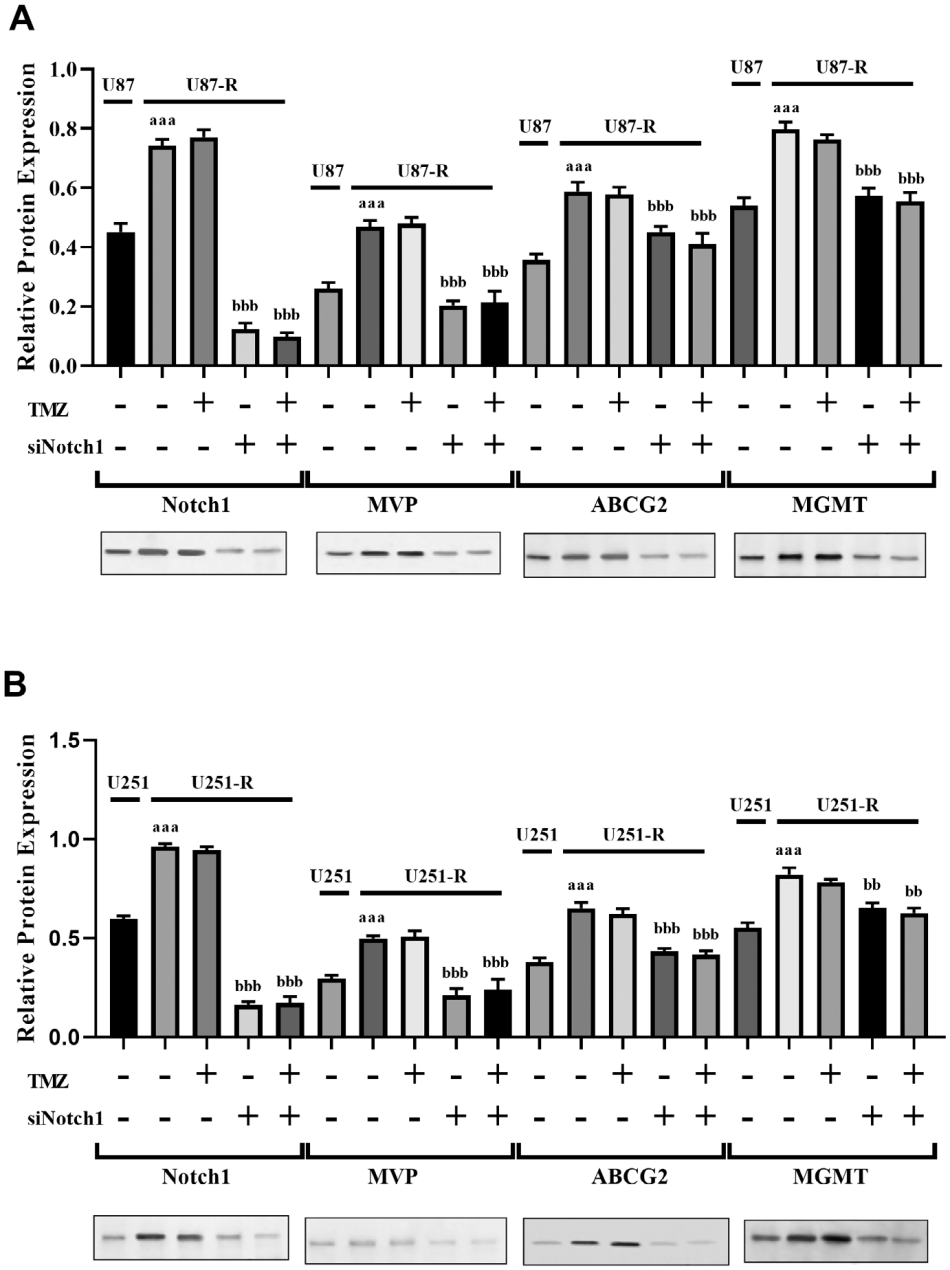
RT‐PCR and Western blot analyzes of multiple resistance proteins in Notch1 knockdown GBM cells. (A) RT‐PCR and Western blot analysis of Notch1, MVP, ABCG2 and MGMT levels in U87 and U87‐R cells, (B) RT‐PCR and Western blot analysis of Notch1, MVP, ABCG2, and MGMT levels in U251 and U251‐R cells. ^aaa^ *p* < 0.0001 compared with TMZ‐non‐resistant GBM cells, ^bb^ *p* < 0.001 compared with TMZ‐resistant GBM cells, and ^bbb^ *p* < 0.0001 compared with TMZ‐resistant GBM cells.

In this study, we assessed whether the knockdown of Notch1 had regulatory effects on MVP, ABCG2, and MGMT in TMZ‐resistant cells treated with TMZ. As shown in Figure 3, TMZ treatment did not lead to significant alterations in the levels of MVP, ABCG2, and MGMT in U87‐R and U251‐R cells (*p* > 0.05). On the other hand, according to ELISA analysis findings, the suppression of Notch1 in U87‐R and U251‐R cells resulted in a notable reduction in the levels of MVP, MGMT, and ABCG2 (*p* < 0.001 and *p* < 0.0001 when compared to TMZ‐resistant groups without TMZ and si‐Notch1 treatment; as depicted in Figure 3A–D). Similarly, si‐Notch1 treatment induced the inhibition of MVP, MGMT, and ABCG2 expression levels in U87‐R and U251‐R cells (*p* < 0.001 and *p* < 0.0001 when compared to TMZ‐resistant groups without TMZ and si‐Notch1 treatment; as shown in Figure 4A,B). Furthermore, consistent with the findings from ELISA and RT‐PCR analyses, Western blot analysis revealed a notable reduction in the protein levels of MVP, MGMT, and ABCG2 after transfecting U87‐R and U251‐R cells with si‐Notch1. Interestingly, it was observed that among the multidrug resistance genes in U87‐R and U251‐R cells, Notch1 downregulation induced the most substantial decrease in MVP levels. As a result, the downregulation of Notch1 led to decreased levels of MVP, ABCG2, and MGMT proteins, increasing the responsiveness of the resistant cells to TMZ.

### 3.3 Notch1 Regulated Epithelial to Mesenchymal Transition (EMT)‐Like Changes in TMZ‐Resistant GBM Cells

**FIGURE 5:**
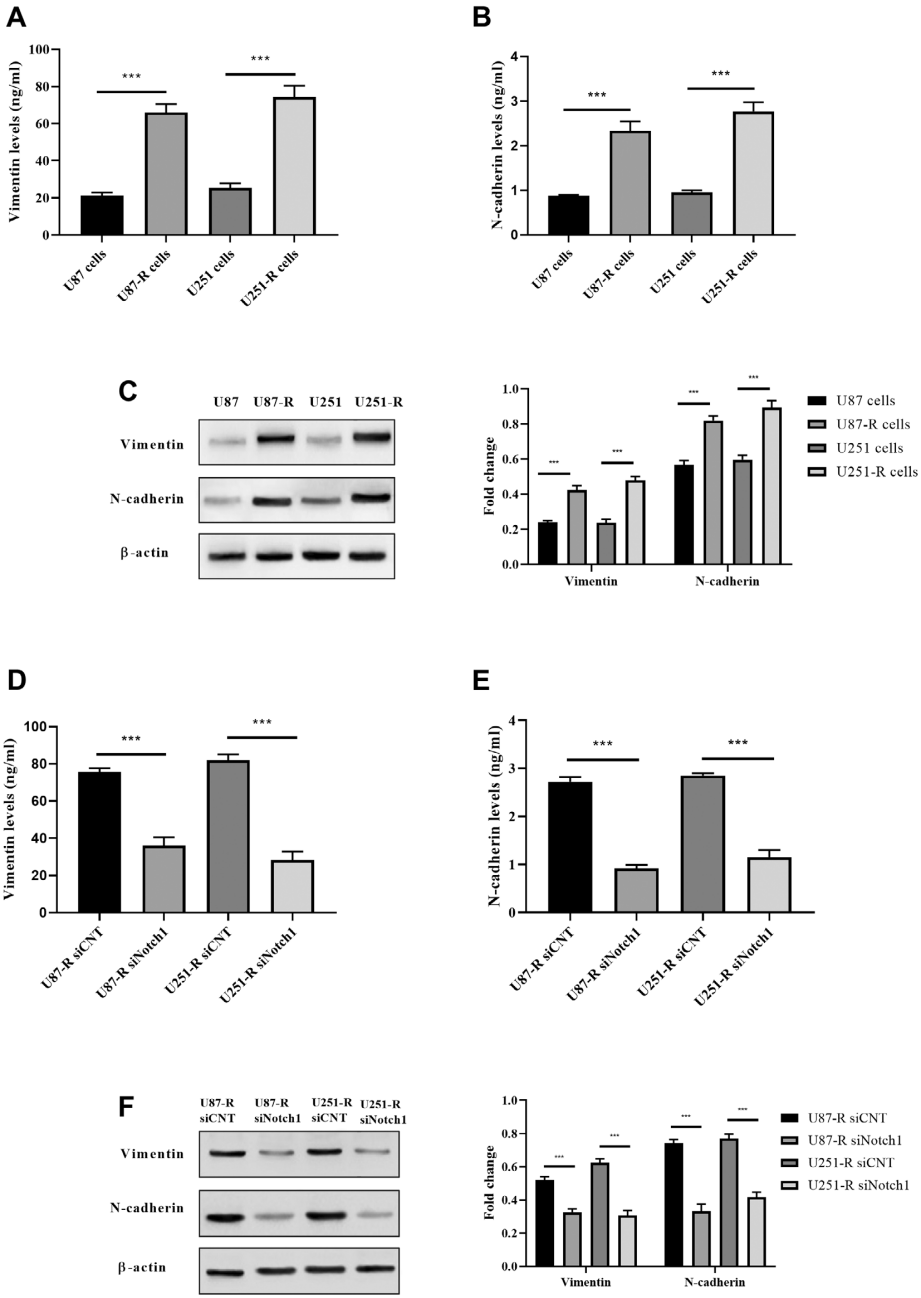
EMT induced in TMZ‐resistant cells was reversed by Notch1 downregulation. (A) ELISA analysis of vimentin levels in TMZ‐resistant and non‐resistant GBM cells, (B) ELISA analysis of N‐cadherin levels in TMZ‐resistant and non‐resistant GBM cells, (C) Western blot analysis of vimentin and N‐cadherin protein levels in TMZ‐resistant and non‐resistant GBM cell, (D) ELISA analysis of vimentin levels in Notch1 knockdown U87‐R and U251‐R cells, (E) ELISA analysis of N‐cadherin levels in Notch1 knockdown U87‐R and U251‐R cells, (F) Western blot analysis of vimentin and N‐cadherin protein levels in Notch1 knockdown U87‐R and U251‐R cells. **p < 0.001, and ***p < 0.0001 compared with control cells.

In our study, we sought to investigate whether the development of TMZ resistance in U87‐R and U251‐R cells was associated with morphological changes, particularly those indicative of epithelial –mesenchymal transition (EMT). As illustrated in Figure 5, we examined whether these resistant cells exhibited molecular alterations characteristic of EMT. In comparison to their parental cells, our analysis via Western blotting revealed an upregulation in the expression of both vimentin and N‐cadherin in U87‐R and U251‐R cells (Figure 5C). Additionally, according to ELISA results, we found that vimentin and N‐cadherin levels enhanced in U87‐R and U251‐R cells (*p* < 0.0001 vs. U87 and U251 cells; Figure 5A,B). These observations strongly suggested that U87‐R and U251‐R cells had acquired EMT‐like properties in contrast to their parental counterparts.

Our findings led us to formulate the hypothesis that Notch1's involvement in the drug resistance phenotype is mediated through the process of epithelial‐mesenchymal transition (EMT). To investigate the potential regulation of EMT by Notch1, we conducted an analysis of EMT markers in U87‐R and U251‐R cells using ELISA and Western blotting. As indicated in Figure 5D–F, the results of this analysis revealed a significant decrease in the expression of N‐cadherin and vimentin, both of which are associated with the EMT process, following the knockdown of Notch1 in U87‐R and U251‐R cells.

## 4 Discussion

GBM represents the most aggressive and poor‐prognosis form of brain tumours. Additionally, it limits surgical success due to its widespread nature in brain tissue. Despite surgical interventions, the prognosis for GBM patients remains exceedingly unfavourable. Resistance to TMZ, a commonly utilised chemotherapy drug, stands as a significant factor contributing to the ineffectiveness of GBM treatment [21]. Additionally, the presence of heterogeneity within GBM further complicates treatment, as the mechanisms behind this heterogeneity and its role in cancer drug resistance are not well understood [22]. There is an urgent need for novel treatment strategies that can effectively target and eliminate scattered tumour cells. Compared with traditional treatments, the development of innovative therapeutic approaches targeting multidrug resistance genes increases treatment effectiveness in cancer, regulates chemosensitivity, and diminishes the potential for metastasis [23]. Our present study has demonstrated a significant overexpression of Notch1 in TMZ‐resistant GBM cells, with this overexpression contributing to their growth. We also investigated whether targeting Notch1 signalling could serve as an effective therapy for TMZ‐resistant GBM. Our findings suggest that the downregulation of Notch1 inhibits the growth of GBM cells, potentially mediated through the deactivation of multidrug resistance genes such as MGMT, ABCG2, and MVP. Moreover, downregulating Notch‐1 expression increased chemosensitivity in GBM cells. These results provide in vitro evidence that Notch‐1 may serve as a viable therapeutic target for TMZ‐resistant GBM cells and hold promise as a candidate for combination therapy with chemotherapy agents.

The Notch signalling serves as a regulatory mechanism to maintain metabolic coordination between pathways related to cell survival, proliferation, differentiation, and apoptosis, as previously indicated [24]. Anomalous activation of Notch signalling has been observed in various cancer types [25]. The dysregulation of the Notch pathway underscores its potential significance in cancer biology. Elevated expression levels of Notch receptors have previously been observed in breast cancers [26]. Furthermore, this research showed that inhibiting Notch‐1 led to the suppression of growth in breast cancer cells by inducing apoptosis, underscoring the oncogenic role of Notch signalling in cancer cells. Although the role of Notch1 signalling in glioma has been the focus of increasing research efforts, its expression in glioblastomas (GBMs) remains a subject of debate. Some reports suggest that Notch1 is highly expressed in GBMs, implying a potential role as a tumour promoter [11, 12]. In contrast, Cheung and colleagues reported the absence of Notch1 in grade IV gliomas, raising the possibility that it may act as a tumour suppressor in different types of tumours [27]. A previous study showed that Notch1 expression was specifically greater in GBM tissue compared to other brain tissues, suggesting that Notch1 may promote proliferation and migration in GBM [28]. Notably, this study also revealed relatively higher levels of Notch1 expression in GBM cells. In alignment with the previously mentioned findings, Guichet et al. provided evidence that the Notch1 signalling pathway regulates the plasticity of GBM stem cells and their angiogenic properties by reducing the expression of neural stem cell transcription factors [29]. Besides, Verhaak and colleagues reported a high expression of Notch signalling in GBM [30]. These collective findings shed light on the complex role of Notch1 in GBMs and its potential as a therapeutic target.

Considering the central role that Notch1 signalling plays in glioma cells, approaches targeting the inhibition of Notch1 hold great promise as a potential avenue for the treatment of GBM. Currently, gamma‐secretase inhibitors (GSIs) function by blocking the final cleavage of the Notch intracellular domain (NICD), effectively inhibiting Notch1 signalling [31]. Results from a phase I trial involving patients with advanced solid tumours treated with the GSI MK‐074250 have shown that this inhibitor can penetrate the blood–brain barrier, target brain‐specific components, and, in some cases, induce complete responses in individuals with malignant glioma [32]. In a separate clinical trial, the therapeutic potential of targeting Notch1 inhibition with GSIs against GBM was evident [33]. However, the persistent challenge of tumour recurrence continues to pose difficulties in GBM treatment. Combining Notch1 inhibition with the targeting of other signalling pathways may yield improved outcomes in clinical trials. In our study, our aim was to investigate whether Notch1 signalling could serve as a therapeutic target for TMZ‐resistant GBM cells by specifically targeting multidrug resistance genes, including MVP, MGMT, and ABCG2. Our findings indicate that the knockdown of Notch‐1 leads to enhanced sensitivity to TMZ, accompanied by reduced expression levels of MGMT and ABCG2. Notably, the impact of downregulating Notch‐1 was particularly pronounced, especially on MVP expression. Additionally, this downregulation of Notch‐1 enhanced the sensitivity of TMZ‐resistant GBM cells to TMZ. Our in vitro results provide evidence that Notch‐1 can be considered a therapeutic target in the treatment of GBM, akin to other multidrug resistance genes. This approach holds promise in addressing TMZ‐resistant GBM and improving treatment outcomes.

In the context of cancer metabolism, elevated levels of MVP were documented in various cancer types [34]. The relationship between increased MVP or vault expression and chemotherapy resistance remains somewhat controversial, with differing viewpoints in the literature [16]. Nevertheless, there exists a widespread consensus that MVP is indeed intricately linked to drug resistance, substantiated by a plethora of studies [35, 36]. MVP serves a critical role in conferring resistance to chemotherapy by facilitating the intracellular transport of drugs into the nucleus and modulating signalling pathways such as MAPK/ERK and phosphoinositide 3‐kinase/Akt [37]. Furthermore, comprehensive investigations conducted by Xiao and colleagues have delved into the mechanisms underlying MVP‐mediated drug resistance [38]. In their research, Noh and colleagues observed an elevation in the expression of Notch1 and MVP in cisplatin‐resistant MDA‐MB‐231 DDPR cells in comparison to their non‐resistant counterparts. Moreover, the levels of Notch1 and MVP proteins exhibited a progressive increase with escalating concentrations of doxorubicin. Notably, when Notch1 was silenced, it resulted in the downregulation of MVP expression. This finding implies that the silencing of Notch1 restored the sensitivity of the cells to cisplatin. Besides, researchers reported that MVP may induce epithelial –mesenchymal transition (EMT), a process contributing to chemoresistance in breast cancer cells. Noh and colleagues conducted an observation revealing an increase in MVP expression within TMZ‐resistant GBM cells [39]. Furthermore, they reported that the downregulation of MVP led to a reduction in TMZ resistance and invasive capacity. Their research suggests a potential negative correlation between MVP expression and the prognosis of GBM patients, implying that elevated MVP levels may be associated with poorer outcomes in individuals with GBM. In our study, we assessed MVP expression in both TMZ‐non‐resistant and TMZ‐resistant cells to investigate its potential correlation with the expression of multidrug‐resistance proteins, including ABCG2 and MGMT. Our findings indicate that the expression levels of ABCG2, MGMT, and MVP are higher in TMZ‐resistant GBM cells. Moreover, we observed that MVP is more affected by Notch1 downregulation than other multidrug resistance proteins.

Until now, the role of Notch1 in relation to chemotherapy responses in GBM cells has remained relatively unexplored. In this study, we demonstrated that the ABCG2, MGMT, and MVP protein levels were notably increased in TMZ‐resistant GBM cells. Additionally, TMZ‐resistant GBM cells exhibited an upregulation of these multidrug resistance proteins along with increased cell proliferation. These findings provide substantial support for the notion of a poor chemotherapy response in the treatment of GBM. Furthermore, a particularly noteworthy observation from our study is that while Notch1 downregulation led to a partial reduction in ABCG2 and MGMT proteins, it had a substantial suppressive effect on MVP. Our study collectively offers valuable insights into the defence mechanisms employed by GBM cells through the specific targeting of Notch1 on ABCG2, MGMT, and, notably, MVP. Consequently, our findings indicate that Notch1 exhibits the potential to overcome TMZ resistance in GBM cells, primarily associated with MVP, while also implicating other genes such as ABCG2 and MGMT. This suggests that targeting Notch1 may hold promise for enhancing the response to TMZ treatment in GBM.

## Author Contributions

**Cengiz Tuncer:** conceptualization (supporting), data curation (supporting), investigation (supporting), methodology (supporting), project administration (supporting), resources (supporting). **Ceyhan Hacioglu:** conceptualization (lead), data curation (lead), formal analysis (lead), funding acquisition (lead), investigation (lead), methodology (lead), project administration (lead), resources (lead), software (lead), supervision (lead), validation (lead), visualization (lead), writing – original draft (lead), writing – review and editing (lead).

## Conflicts of Interest

The authors declare no conflicts of interest.

## Data Availability

The dataset is generated from the corresponding author on reasonable request.
